# Total hip arthroplasty, combined with a reinforcement ring and posterior column plating for acetabular fractures in elderly patients: good outcome in 34 patients

**DOI:** 10.1080/17453674.2019.1597325

**Published:** 2019-04-01

**Authors:** Tõnis Lont, Jyrki Nieminen, Aleksi Reito, Toni-Karri Pakarinen, Ilari Pajamäki, Antti Eskelinen, Minna K Laitinen

**Affiliations:** a Coxa Hospital for Joint Replacement, Tampere, Finland;;; b Department of Orthopaedics and Traumatology, Unit of Musculoskeletal Surgery, Tampere University Hospital, Tampere, Finland;;; c Department of Orthopaedics and Traumatology, Helsinki University Hospital, Helsinki, Finland

## Abstract

Background and purpose — Low-energy acetabulum fractures are uncommon, and mostly occur in elderly patients. Determining the optimal operative treatment for such fractures is challenging. Here we investigated whether acutely performed total hip arthroplasty plus posterior column plating (THA) reduced complications and reoperations compared with open reduction and internal fixation (ORIF) in elderly patients with acetabular fractures.

Patients and methods — We retrospectively reviewed the records of 59 patients, > 55 years of age, with complex acetabular fractures, caused by low-energy trauma, treated between January 2008 and September 2017. Of these patients, 34 underwent acute THA, and 25 ORIF alone. Patient and implant survival were compared between groups using Kaplan–Meier survival analysis and Cox multiple regression. Functional outcomes assessed by Oxford Hip Score (OHS) were compared between the THA patients and those 9 ORIF patients who underwent secondary THA due to posttraumatic hip osteoarthritis (OA) during follow-up.

Results — Overall patient survival was 90% (95% CI 82–98) at 12 months, and 64% (CI 47–81) at 5 years. Of 25 ORIF patients, 9 required secondary THA due to posttraumatic OA. Large fragments on the weight-bearing acetabular dome upon imaging predicted ORIF failure and secondary THA. The acute THA group and secondary THA group had similar 12-month OHS.

Interpretation — Acute THA including a reinforcement ring resulted in fewer reoperations than ORIF alone in elderly patients with acetabular fractures. These findings support acute THA as first-line treatment for complex acetabular fractures in elderly patients.

Low-energy acetabulum fractures are rare, and mostly occur in elderly patients with comminuted and complex fracture patterns. They are associated with unfavorable prognostic signs, such as articular impaction and fragmentation, pre-existing osteoarthritis (OA) of the hip, and osteopenia (Anglen et al. 2003, Laflamme et al. 2011). Acetabular fracture treatment depends on the fracture type as well as on patient-related factors, such as comorbidities. Treatment options in elderly patients include a nonoperative approach, open reduction and internal fixation (ORIF), and acute total hip arthroplasty (THA) with or without simultaneous ORIF (Daurka et al. 2014).

Nonoperative treatment is optimal for non-displaced fractures and in patients with severe comorbidities (Guerado et al. 2012). Surgery for low-energy displaced acetabular fractures is challenging. Poor bone quality may impede stable osteosynthesis. Poor prognostic indicators for ORIF include anteromedial dome impaction, poor reduction and fixation of the weight-bearing dome, and associated pelvic fractures (Anglen et al. 2003, Laflamme et al. 2011). Recent reports describe promising results with the combination of THA and ORIF (Boelch et al. 2017, Ortega-Briones et al. 2017, Salama et al. 2017). However, no study has compared the outcomes of these different treatment options; the optimal treatment for low-energy comminuted acetabular fractures in the elderly population remains unclear.

In this retrospective study we examined whether acutely performed THA including posterior column plating would result in fewer complications and reoperations than ORIF alone in elderly patients with comminuted acetabular fractures. In addition we compared functional outcome Oxford Hip Score between patients healed by THA and patients healed with secondary THA after a failed ORIF.

## Patients and methods

We retrospectively reviewed the records of all patients over 55 years of age who were diagnosed with and treated for a low-energy comminuted acetabular fracture at our hospitals between January 1, 2008, and September 1, 2017. Patients were identified from a prospectively maintained database that identifies and records all patients referred to and managed in the unit. The study population comprised 59 patients, of whom 25 were treated with ORIF alone and 34 with acute THA including posterior column plating. There were no definitive radiographic criteria for the treatment of patients with either ORIF or THA. Patients who underwent ORIF were treated at the Tampere University Hospital, Tampere, Finland. All patients who underwent THA were operated on at the Coxa Hospital for Joint Replacement, Tampere, Finland, which performs all arthroplasties in the same region. Both hospitals are located in the same building and share the same emergency room. Acute trauma patients are discussed and treatment decisions are made in a collective meeting pragmatically by treating physicians.

**Figure 1.**
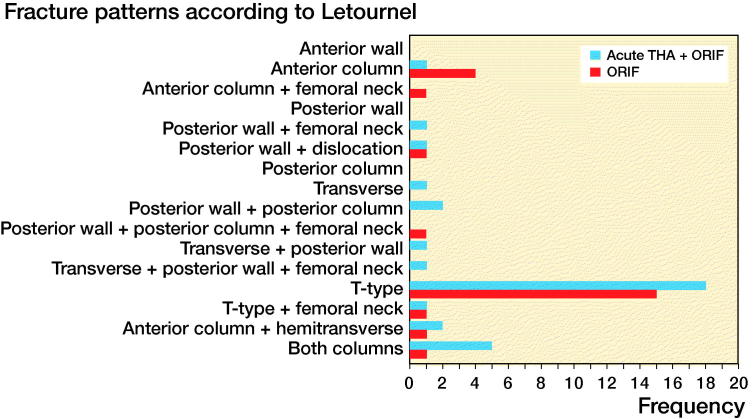
Fracture patterns according to Letournel classification.

Fracture patterns according to Letournel classification were determined based on preoperative radiographs (Figure 1). The majority of patients exhibited a complex T-type fracture with central femoral head protrusion (Figures 2). Patient demographics were similar between the treatment groups, except that follow-up was longer in the ORIF group than in the THA group. Based on the Charlson Comorbidity Index (CCI), patients in the THA group tended to have more severe comorbidities than patients in the ORIF group, but this difference was not statistically significant (median CCI of 5 vs. 4; p = 0.1). A causal directed acyclic graph was used to investigate confounding factors (Figure 3, see Supplementary data). Table 1 summarizes the patient characteristics.

Figure 2.A. 75-year-old patient with T-type fracture and central protrusion of the femoral head. Red line indicates the Gull sign. B. Computed tomography showing quadrilateral surface comminution and central protrusion of femoral head. C. The same patient at last follow up. The femoral head had been morsellized and used as filling

**Figure:**
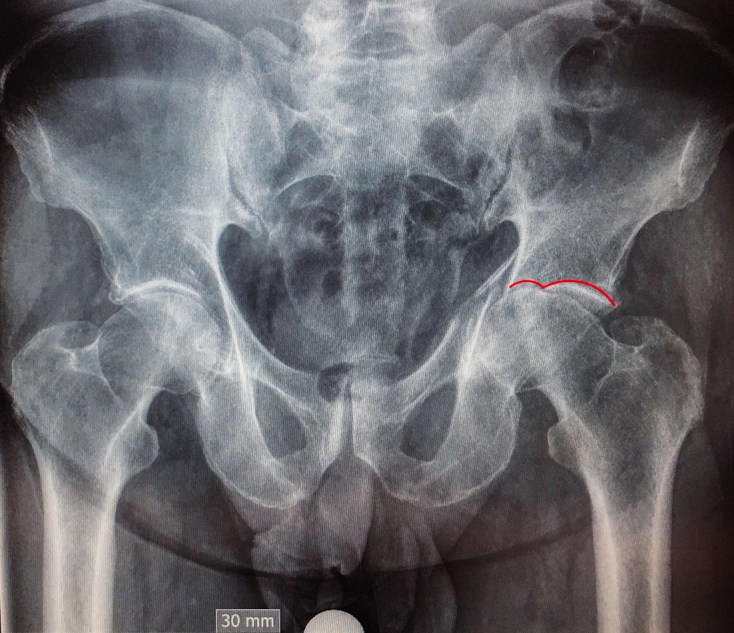

**Figure:**
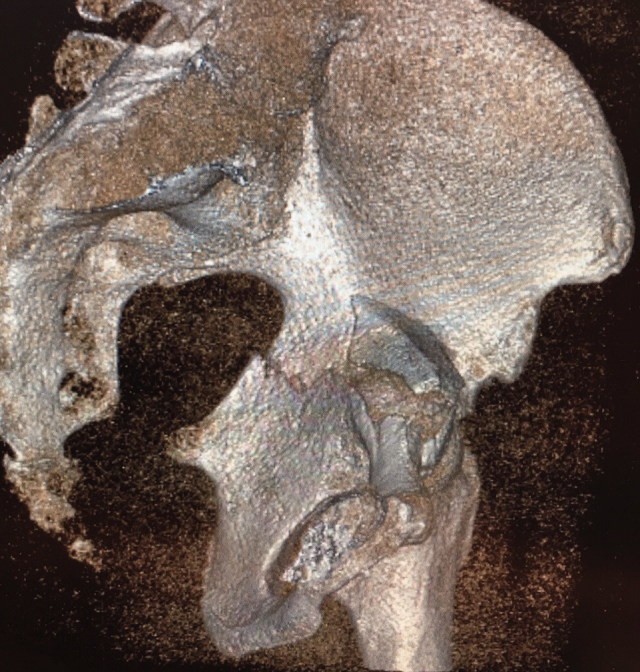

**Figure:**
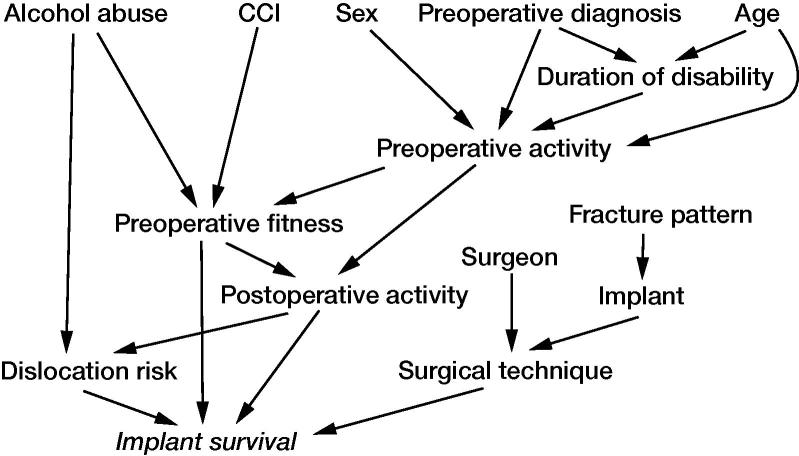

Figure 3.Postoperative patient survival stratified by surgical group.

**Figure:**
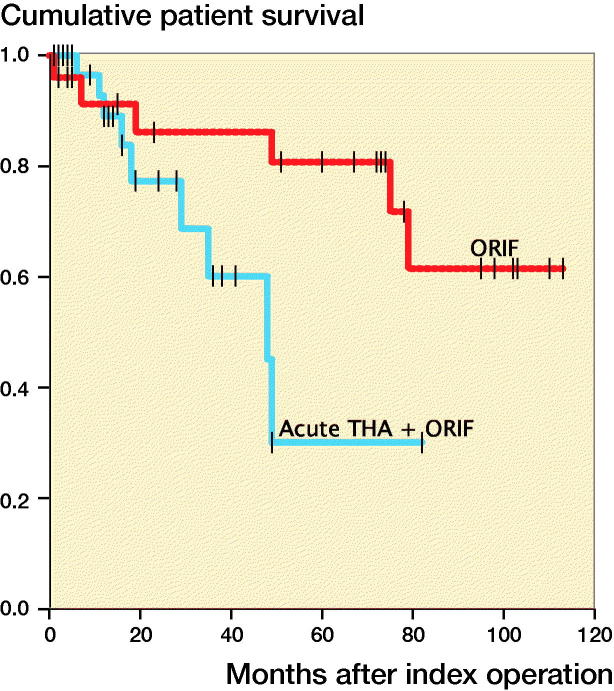

**Figure:**
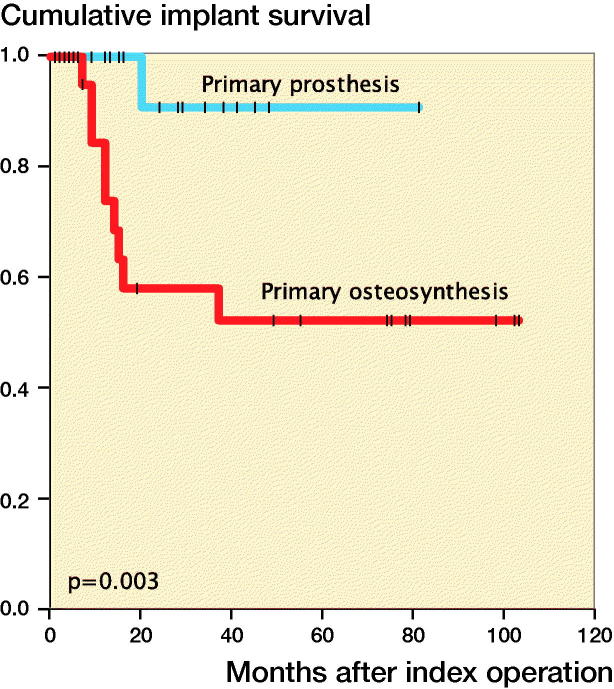

**Table 1.** Patient demographics. Values are mean (range) unless otherwise specified

		Primary operative strategy		
Characteristic	Total	THA + ORIF	ORIF only	p-value
Number	59	34	25	
Age	70 (56–92)	71 (56–92)	69 (58–83)	0.2
Follow-up, years	2.6 (0–9)	1.4 (0–6)	4.2 (0–9)	0.01
BMI	26 (15–42)	25 (15–42)	26 (20–36)	0.2
Women, n	17	10	7	0.4
Estimated blood loss, L	1.2 (0.4–2.7)	1.1 (0.4–2.7)	1.4 (0.3–3.7)	0.4
Operating time, min	190 (97–321)	169 (97–310)	218 (120–321)	0.01
Stem, n				0.01
Cementless	22	13	9	
Cemented	21	13		
Acetabular component, n				0.01
Cemented	3	3	0	
Cemented constrained	34	31	3	
Cementless	6	0	6	
Heart disease, n	14	8	6	0.6
Neurologic disease, n	10	7	3	0.3
Alcohol abuse, n	6	5	1	0.2
Diabetes, n	13	7	6	0.5
Trauma, n				0.5
Falling from the same level	45	25	20	
Fall from high	3	2	1	
Motor vehicle accident	6	5	1	
Biking	5	2	3	
Complications, n				0.01
Secondary OA leading to THA		–	9	
Dislocation		1	–	
Periprosthetic fracture		1	–	
Infection		0	1	
CCI, median	5	5	4	0.1

THA: total hip arthroplasty; ORIF: open reduction and internal fixation;

BMI: body mass index; OA: osteoarthritis; CCI: Charlson Comorbidity Index

ORIF procedures were performed by senior pelvic trauma surgeons. Patients were administered preoperative antibiotic prophylaxis, and placed under general anesthesia. Surgery was initiated with the patient in a supine position. An anterior intrapelvic (AIP) approach was used through a low midline incision. When necessary, a lateral window/first window of an ilioinguinal approach was used to fix the anterior and lateral parts of the pelvis. Thereafter, the patient was repositioned into the lateral decubitus position, and a Kocher–Langenbeck approach was used to access the posterior acetabular fracture components, when necessary.

Acute THA was performed by experienced revision arthroplasty surgeons together with pelvic trauma surgeons. Patients were placed under spinal anesthesia, and a dose of preoperative antibiotic prophylaxis was infused 30 min prior to surgery. A Kocher–Langenbeck approach was used. The posterior column was supported by adding posterior column plating and a GAP II reinforcement ring (Stryker, Mahwah, NJ, USA). Various components were used in both the femur and acetabulum throughout the study period, depending on the implant selected by the hospital (Table 1). In all acute THA cases, morselized autograft bone transplantation from the resected femoral head was performed using an impaction grafting technique (Hosny et al. 2017). In the first acute THA case, anterior column reduction and fixation was performed using an AIP approach. Additional anterior fixation was not applied in any subsequent patients.

Thromboprophylaxis started at 6 hours postoperatively and continued until 4 weeks postoperatively. Mobilization was started on the first postoperative day. ORIF-treated patients were mobilized with partial-weight-bearing walking aids for 6 weeks, after which full weight-bearing was allowed. For patients who underwent acute THA, the goal was partial weight-bearing for 6 weeks, but if patients were unable to follow restrictions, weight-bearing was allowed as tolerated.

During follow-up visits, patients were clinically evaluated and subjected to pelvic radiographs. The OHS was administered at outpatient clinic visits, or via a routine letter request sent to patients with THA at 12 months. All complications encountered during follow-up were recorded.

### Statistics

Patient and implant survival rates were assessed using the Kaplan–Meier method. Between-group comparisons were performed using the log-rank test. The Cox regression model was used to identify independent factors affecting patient survival. Continuous variables were reported as mean and 95% confidence interval (CI), and compared between groups by t-test. Differences in proportions were assessed using Fisher’s exact test. Follow-up time was calculated from the date of surgery to the date of the most recent revision, follow-up, or death. We calculated the CI for relative risks. All analyses were performed using SPSS Statistics 24.0 (IBM Corp, Armonk, NY, USA), and a p-value of < 0.05 was considered significant.

### Ethics, funding, and potential conflicts of interest

This retrospective study was approved by the local chair of the audit department. The study was funded by the Competitive Research Funding of Tampere University Hospital. No competing interests are declared.

## Results

Overall patient survival after fracture was 90% (CI 82–98) at 12 months, and 58% (CI 38–77) at 5 years. In the ORIF group, 12-month survival was 91% (CI 73–100) and 5-year survival was 86% (CI 63–87). In the acute THA group, the respective survival rates were 89% (CI 77–100) and 30% (CI 2–62) (Figure 3). Univariable analysis revealed that mortality was statistically significantly associated to some extent with low CCI, neurological disease, and alcoholism. In multiple regression analysis, all predictors showed plausible effects on mortality, but the null hypothesis could not be rejected based on the HRs and their corresponding CIs.

**Figure 4.**
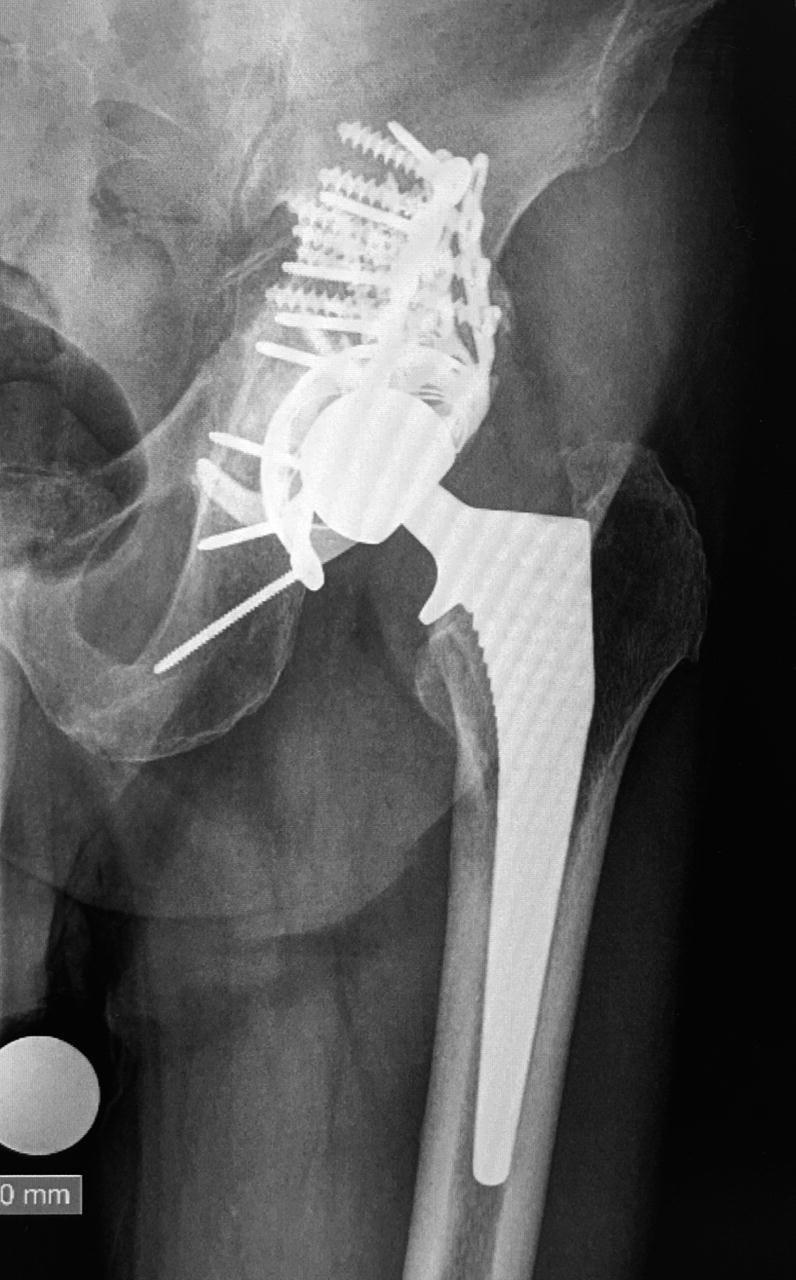
Implant survival stratified by surgical group.

Of the 25 ORIF patients, 9 developed posttraumatic OA necessitating secondary THA at a median of 12 months (7–37) after fracture surgery. Kaplan–Meier analysis revealed that implant survival was 74% (CI 54–94) at 12 months and 52% (CI 30–75) at 2 years in the ORIF group, and was 100% at 12 months and 91% (CI 74–108) at 2 years in the acute THA group (Figure 4). After adjustment for other variables, ORIF was associated with a hazard ratio (HR) of 12. However, the 95% confidence interval indicated that the data were consistent with a wide range of plausible HRs, from 1.4 to 91.

All ORIF-treated patients showed good reduction quality, with a gap of less than 2–3 mm. Patients with unsuccessful ORIF tended to show a gull/gull-wing radiological sign and a considerable fragment in the acetabular weight-bearing area dome, which was observed in 6 of the 9 patients requiring secondary THA. Among the 16 patients successfully treated with ORIF alone, 2 also exhibited a fragment in the weight-bearing dome. The follow-up duration was shorter in the acute THA group than in the ORIF group, with a mean difference in follow-up time of 3 months (CI 2–4) (Table 2). Radiographic follow-up revealed that all fractures healed, and the acetabular autologous bone grafts were well incorporated in patients in the acute THA group (Figure 2C).

**Table 2.** Comparison of patients treated with acute THA + ORIF and those treated by ORIF alone followed by secondary THA due to posttraumatic OA. Values are median (range) unless otherwise specified

	Acute THA + ORIF	ORIF with secondary THA	Mean difference	p-value
Number	34	9		
Age	70 (56–87)	65 (58–74)	3	0.1
Follow-up, months	15 (1–82)	72 (15–113)	3	0.0
BMI	23 (15–42)	28 (21–33)	1	0.4
Women, n	10	3		1
Estimated blood loss, L	1.1 (0.4–2.7)	1.1 (0.700–2.0)	60	0.8
Operating time, min	169 (97–310)	143 (100–269)	26	0.2
Oxford Hip Score at 12 months	41 (33–46)	42 (6–48)	2	0.3
Trauma, n				0.4
Falling from the same level	25	6		
Fall from high	2	–		
Motor vehicle accident	5	1		
Biking, n	2	2		
Complications, n				0.2
Infection	–	2		
Dislocation	1	–		
Periprosthetic fracture	1	–		

For abbreviations, see Table 1.

Between groups, OHD differed by a mean of 2.1 points (CI −2.4 to 6.6). The OHS exceeded 37 points in 75% of patients. In the acute THA group, 2 patients developed complications: 1 patient had a periprosthetic fracture leading to femoral component revision, and 1 patient had recurrent dislocations that were treated with closed reductions. In the secondary THA group, 2 patients developed deep infections leading to two-stage revision arthroplasty. These groups are compared in Table 2.

## Discussion

Osteoporotic fragility fractures, especially in the pelvic area, are increasingly common as the elderly population grows (Kim et al. 2015, Rinne et al. 2017). Fragility fractures are more frequently comminuted, and these fractures and low bone quality are associated with poor outcomes with ORIF; thus, the operative strategy differs from that used in younger patients with good bone quality (Anglen et al. 2003, Ferguson et al. 2010, Salama et al. 2017). Recently, the more straightforward approach of acute THA combined with ORIF has gained popularity as an alternative to tedious and time-consuming fracture reduction and internal fixation with osteosynthesis (Boelch et al. 2017, Ortega-Briones et al. 2017, Salama et al. 2017). In our retrospective study, we compared ORIF and acute THA for the treatment of osteoporotic acetabular fractures in elderly patients. We found that acute THA including posterior column plating with a reinforcement ring resulted in a low complication rate and carried a low risk for revision. This appears to be a safe procedure in elderly patients with comminuted low-energy fractures of the acetabulum. ORIF alone carried a relatively high risk of posttraumatic OA necessitating a secondary THA.

To our knowledge, this is the first study to directly compare the two most common treatment options for these fractures. The reasons to choose acute THA over more conventional osteosynthesis may not be obvious. Risk factors for osteosynthesis failure are poorly defined in the literature, but generally include marginal impaction, femoral head damage, severe fracture comminution, and the presence of a gull sign (Anglen et al. 2003, Kreder et al. 2006, Gary et al. 2011, Daurka 2014, Li and Tang 2014). In our study, fragment impaction in the acetabular weight-bearing-area dome was a sign of an unfavorable prognosis in ORIF-treated patients, which is in accordance with current literature (Anglen et al. 2003, Laflamme et al. 2011). A large fragment in the anteromedial dome was detected upon imaging in 6 of the 9 patients who were initially treated with ORIF and subsequently required secondary THA. The overall rate of secondary THA in the ORIF group was one-third in our study, which is much higher than the 10% rate reported in the literature. However, prior reports have usually included young patients or all fracture types (Tannast et al. 2012). Among elderly patients, the rate of secondary THA after ORIF widely varies from 19% up to as high as 100% in elderly patients with fractures exhibiting femoral head or acetabular impaction. Elderly patients infrequently have both femoral head and acetabular impaction (Kreder et al. 2006, Archdeacon et al. 2013, Clarke-Jenssen et al. 2017). The high rate of secondary THA in our study was likely due to the comminuted fracture patterns seen in our elderly patients. One major issue with ORIF is the prolonged restriction on weight-bearing. Some elderly patients do not comply with the weight-bearing restriction, resulting in either failures or permanent immobilization (Archdeacon et al. 2013).

Patients had a median age of 70, but there were relatively few complications and none leading to perioperative or immediate postoperative death. The rate of infection was generally low, but was higher in the secondary THA group compared with the acute THA group. The rate of dislocation was also surprisingly low in all patients. Only 1 patient in the acute THA group had a recurrent dislocation, which was not revised because the patient evidently abused alcohol. Only the first patient in the acute THA group underwent plate fixation of both the anterior and posterior columns. Increasing evidence supports the practice of using only posterior plating (Anglen et al. 2003, Carroll et al. 2010, Herscovici et al. 2010, Salama et al. 2017). After the first patient, the remaining acute THA cases received plate fixation of only the posterior column, resulting in reduced operation times, an easier approach, and simplified postoperative rehabilitation that diminished postoperative complications. There were no revisions or complications due to instability, supporting the hypothesis that posterior column plating was sufficient to stabilize the pelvis in these patients. Our findings and the current literature emphasize the importance of posterior column stabilization and prevention of central migration during treatment using a posterior column plate and an anti-protrusion cage. It remains unclear why the anterior column does not require fixation (Herscovici et al. 2010, Guerado et al. 2012, Rickman et al. 2012, Boelch et al. 2017, Ortega-Briones et al. 2017, Salama et al. 2017).

Our study has several limitations, including those inherent to its retrospective non-randomized design. Additionally, the number of patients is small, limiting the statistical power of our analyses. Mobility and functional scores were not preoperatively assessed. The results may have also been influenced by the short-term follow-up for the acute THA group. However, a properly conducted survivor analysis that accounted for follow-up time revealed a decreased failure rate in the acute THA group.

The majority of injuries in the present study were caused by low-energy falls from the same level. There were also several injuries involving low-speed bicycle and motor vehicle accidents, but these injuries were mostly caused by patient confusion. The population was most similar to the hip fracture population (Panula et al. 2011), but with an improved overall survival of 90% at 12 months, decreasing to 64% at 5 years. This improved survival might be biased because most fragile patients with acetabular fractures are not considered for operative treatment, and were thus excluded from our study. During the first postoperative year, survival was equal between the 2 treatment groups. Subsequently, the survival rate exhibited a more rapid decrease in the acute THA group compared with the osteosynthesis group, although this difference was not statistically significant. The retrospective design of this study reveals differences in patients between the treatment groups, but also demonstrates the pragmatic decision-making indicated by the CCI. Patients in the acute THA group had more comorbidities, and thus required a long-lasting surgical procedure that avoided the high revision rate in the ORIF group.

The OHS is a validated, reliable, and well-established assessment tool for evaluating the outcome of THA (Dawson et al. 1996). In our study, OHS differed between treatment groups by a mean of 2 points, and did not differ between the acute THA group and the secondary THA group. In a recent study, Hamilton et al. (2018) defined “treatment success” following THA based on an OHS threshold value of 37.5 points, since over 90% of THA patients with an OHS value of over 37.5 points expressed satisfaction with the surgical outcome (Hamilton et al. 2018). In our study, 75% of patients had an OHS exceeding this “treatment success” level, which can be regarded as a good outcome in this older and fragile patient group.

In conclusion, our present results demonstrated that acute THA, performed simultaneously with a reinforcement ring and ORIF, resulted in fewer reoperations, improved implant survival, and yielded a good functional outcome when compared with ORIF alone in elderly patients with complex osteoporotic acetabular fractures. We prefer this acute THA procedure as first-line treatment in this patient population, especially when preoperative radiographs reveal a large dome fragment. However, this surgery is complex and requires a multidisciplinary team with a mixed skill set.

### Supplementary data

Figure 3 is available as supplementary data in the online version of this article, http://dx.doi.org/10.1080/17453674.2019.1597325

The present study was planned and designed by TL, JN, and MKL. Statistical analyses were performed by AR, TL, and MKL. The manuscript was written by TL and MKL. All authors participated in the data interpretation, and the critical revision of the manuscript. 

*Acta* thanks Olav Røise and Morten Schultz Larsen for help with peer review of this study.

## Supplementary Material

Supplemental Material

## Supplementary data

Figure 3 is available as supplementary data in the online version of this article, http://dx.doi.org/10.1080/17453674.2019.1597325

## References

[CIT0001] Anglen J O, Burd T A, Hendricks K J, Harrison P The “Gull Sign”: a harbinger of failure for internal fixation of geriatric acetabular fractures. J Orthop Trauma 2003; 17(9): 625–34. 14574190 10.1097/00005131-200310000-00005

[CIT0002] Archdeacon M T, Kazemi N, Collinge C, Budde B, Schnell S Treatment of protrusio fractures of the acetabulum in patients 70 years and older. J Orthop Trauma 2013; 27(5): 256–61. 318269126f. 22810547 10.1097/BOT.0b013e318269126f

[CIT0003] Boelch S P, Jordan M C, Meffert R H, Jansen H Comparison of open reduction and internal fixation and primary total hip replacement for osteoporotic acetabular fractures: a retrospective clinical study. Int Orthop 2017; 41(9): 1831–7. 27511470 10.1007/s00264-016-3260-x

[CIT0004] Carroll E A, Huber F G, Goldman A T, Virkus W W, Pagenkopf E, Lorich D G, Helfet D L Treatment of acetabular fractures in an older population. J Orthop Trauma 2010; 24(10): 637–44. 20871252 10.1097/BOT.0b013e3181ceb685

[CIT0005] Clarke-Jenssen J, Roise O, Storeggen S A O, Madsen J E Long-term survival and risk factors for failure of the native hip joint after operatively treated displaced acetabular fractures. Bone Joint J 2017; 99-b(6): 834–40. 28566406 10.1302/0301-620X.99B6.BJJ-2016-1013.R1

[CIT0006] Daurka J S, Pastides P S, Lewis A, Rickman M, Bircher M D Acetabular fractures in patients aged > 55 years: a systematic review of the literature. Bone Joint J 2014; 96-B(2): 157–63. 24493178 10.1302/0301-620X.96B2.32979

[CIT0007] Dawson J, Fitzpatrick R, Carr A, Murray D Questionnaire on the perceptions of patients about total hip replacement. J Bone Joint Surg Br 1996; 78(2): 185–90. 8666621

[CIT0008] Ferguson T A, Patel R, Bhandari M, Matta J M Fractures of the acetabulum in patients aged 60 years and older: an epidemiological and radiological study. J Bone Joint Surg Br 2010; 92(2): 250–7. 20130318 10.1302/0301-620X.92B2.22488

[CIT0009] Gary J L, Lefaivre K A, Gerold F, Hay M T, Reinert C M, Starr A J Survivorship of the native hip joint after percutaneous repair of acetabular fractures in the elderly. Injury 2011; 42(10): 1144–51. 20850738 10.1016/j.injury.2010.08.035

[CIT0010] Guerado E, Cano J R, Cruz E Fractures of the acetabulum in elderly patients: an update. Injury 2012; 43(Suppl 2): S33–S41. 23622990 10.1016/S0020-1383(13)70177-3

[CIT0011] Hamilton D F, Loth F L, MacDonald D J, Giesinger K, Patton J T, Simpson A H, Howie C R, Giesinger J M Treatment success following joint arthroplasty: defining thresholds for the Oxford Hip and Knee Scores. J Arthroplasty 2018; 33(8): 2392–7. 29691169 10.1016/j.arth.2018.03.062

[CIT0012] Herscovici D Jr, Lindvall E, Bolhofner B, Scaduto J M The combined hip procedure: open reduction internal fixation combined with total hip arthroplasty for the management of acetabular fractures in the elderly. J Orthop Trauma 2010; 24(5): 291–6. 20418734 10.1097/BOT.0b013e3181b1d22a

[CIT0013] Hosny H A H, El-Bakoury A, Fekry H, Keenan J Mid-term results of Graft Augmentation Prosthesis II cage and impacted allograft bone in revision hip arthroplasty. J Arthroplasty 2017. 10.1016/j.arth.2017.11.060 29310917

[CIT0014] Kim J W, Herbert B, Hao J, Min W, Ziran B H, Mauffrey C Acetabular fractures in elderly patients: a comparative study of low-energy versus high-energy injuries. Int Orthop 2015; 39(6): 1175–9. 25728534 10.1007/s00264-015-2711-0

[CIT0015] Kreder H J, Rozen N, Borkhoff C M, Laflamme Y G, McKee M D, Schemitsch E H, Stephen D J Determinants of functional outcome after simple and complex acetabular fractures involving the posterior wall. J Bone Joint Surg Br 2006; 88(6): 776–82. 16720773 10.1302/0301-620X.88B6.17342

[CIT0016] Laflamme G Y, Hebert-Davies J, Rouleau D, Benoit B, Leduc S Internal fixation of osteopenic acetabular fractures involving the quadrilateral plate. Injury 2011; 42(10): 1130–4. 21156315 10.1016/j.injury.2010.11.060

[CIT0017] Li Y L, Tang Y Y Displaced acetabular fractures in the elderly: results after open reduction and internal fixation. Injury 2014; 45(12): 1908–13. 25267400 10.1016/j.injury.2014.09.004

[CIT0018] Ortega-Briones A, Smith S, Rickman M Acetabular fractures in the elderly: midterm outcomes of column stabilisation and primary arthroplasty. BioMed Res Int 2017; 2017:4651518. 28194414 10.1155/2017/4651518 PMC5282405

[CIT0019] Panula J, Pihlajamaki H, Mattila VM, Jaatinen P, Vahlberg T, Aarnio P, Kivela S L Mortality and cause of death in hip fracture patients aged 65 or older: a population-based study. BMC Musculoskeletal Disorders 2011; 12: 105. 21599967 10.1186/1471-2474-12-105 PMC3118151

[CIT0020] Rickman M, Young J, Bircher M, Pearce R, Hamilton M The management of complex acetabular fractures in the elderly with fracture fixation and primary total hip replacement. Eur J Trauma Emerg Surg 2012; 38(5): 511–6. 26816252 10.1007/s00068-012-0231-9

[CIT0021] Rinne P P, Laitinen M K, Huttunen T, Kannus P, Mattila V M The incidence and trauma mechanisms of acetabular fractures: a nationwide study in Finland between 1997 and 2014. Injury 2017; 48(10): 2157–61. 28807429 10.1016/j.injury.2017.08.003

[CIT0022] Salama W, Mousa S, Khalefa A, Sleem A, Kenawey M, Ravera L, Masse A Simultaneous open reduction and internal fixation and total hip arthroplasty for the treatment of osteoporotic acetabular fractures. Int Orthop 2017; 41(1): 181–9. 27020781 10.1007/s00264-016-3175-6

[CIT0023] Tannast M, Najibi S, Matta J M Two to twenty-year survivorship of the hip in 810 patients with operatively treated acetabular fractures. J Bone Joint Surg Am 2012; 94(17): 1559–67. 22992846 10.2106/JBJS.K.00444

