# Supplementary material for: Total hip arthroplasty, combined with a reinforcement ring and posterior column plating for acetabular fractures in elderly patients: good outcome in 34 patients
Source: Acta Orthop. 2019 Apr 1;90(3):275–80. doi: 10.1080/17453674.2019.1597325 (PMC6534240; doi:10.1080/17453674.2019.1597325)
Supplement: Supplemental Material [file IORT_A_1597325_SM6241.pdf]

Supplementary data

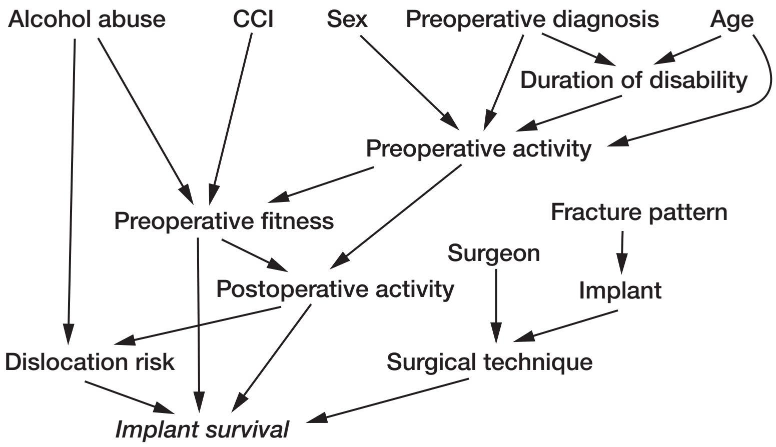

Figure 3. Causal pathways in directed acyclic graphs in the variable selection. *Exposure of interest* = Implant, *Outcome* = Implant survival, *Suggested covariates* = Sex, age, alcohol abuse, Charlson Comorbidity Index (CCI), preoperative diagnosis (top row)
